# The Methods of Examining the Stomach

**Published:** 1899-04-01

**Authors:** 


### Hospital Clinics and Medical Progress.

### THE METHODS OF EXAMINING THE STOMACH.

A Postgraduate Lecture delivered at the West London Hospital by Leonard A. Bidwell, F.R.C.S., Senior Assistant Surgeon to the Hospital.

The following are the methods of examining the stomach:—(1) inspection, (2) palpation, (3) percussion, (4) auscultation, (5) intubation, (6) artificial distension with gas, (7) the gastric diaphane, (8) chemical examination of its contents, and (9) exploratory laparotomy. Before describing these methods, I would remind you of the normal limits of the stomach—they are as follows: The lower limit corresponds to a curved line lying midway between the umbilicus and the end of the sternum, and commencing at the tenth costal cartilages; the upper limit extends to the level of the lower border of the fifth rib on the left side.

By inspection peristaltic waves may be seen in the neighbourhood of the pylorus in cases of pyloric stenosis, these waves will pass from left to right; when the stomach is dilated, a tumour may be seen in the umbilical region, or it may occupy nearly the whole of the abdominal cavity.

Palpation is an important method of examining the stomach, and should be done with the patient lying on his back with the knees drawn up; the right hand, previously warmed, should be placed flat on the abdomen just above the umbilicus, then by gentle but steady pressure the resistance of the abdominal muscles will be overcome, and by drawing the fingers up the deeper parts will be felt with the tips of the fingers; in examining a fresh part of the abdomen the hand should not be removed, but should be made to slide over the skin; to facilitate the examination the patient's attention should be distracted by making him talk about other symptoms. By these means any swelling which is present will be detected, and by attempting to glide the abdominal walls over it you will be able to satisfy yourself (1) whether the swelling is in the abdominal wall or beneath it, (2) whether the abdominal parietes are adherent to the swelling, and (3) whether the tumour is freely moveable. In cases of solid tumours, with a lax abdominal wall, it will be possible to grasp the edge of the tumour itself. Further, the existence of peritoneal adherence may sometimes be diagnosed by a curious creaking sensation.

Percussion is also a valuable aid to diagnosis; by it we can accurately define the limits of the stomach when empty and when artificially distended. When, however, the stomach is full, a duller note will be obtained in the lower part, caused by the presence of liquid; another difficulty is that the note of the colon is often identical with that of the stomach, and when a growth is present a dull note will be obtained over its area. The upper limit of the stomach is always easy to define; it will, however, often be found that the upper limit of the stomach is below the costal arch in cases of dilatation and of gastroptosis.

Auscultation affords valuable information in many cases of dilatation of the stomach from pyloric stenosis, since we shall hear bubbling of gas in the pyloric region, and by placing the stethoscope over the stomach wall and tapping at a distant spot we shall hear loud splashing sounds; we may also hear crepitations when adhesions are present. To obtain more accurate information we may have to practice intubation or artificial distension with gas, or introduce a gastric diaphane.

In intubation, a soft rubber stomach tube should always be used, since this is passed with very little discomfort to the patient. It is quite unnecessary to place a finger in the patient's mouth, but all that is required is to pass the end to the posterior wall of the pharynx, and then direct the patient to swallow. The tube will then be carried by the muscles of deglutition into the commencement of the œsophagus. It can be gently pushed on into the stomach. To evacuate the contents, either a symphon may be attached to end of the stomach tube, or the contents may be expressed by steady pressure over the epigastric region. The latter is the better method.

Artificial distension of the stomach by gas is effected either by pumping in air through the stomach tube, or, if this has not been passed, by giving a small seidlitz powder separately. First the bicarbonate of soda is given in half a tumbler of water, followed immediately by the tartaric acid also dissolved in water. The carbonic acid gas, generated in this way, causes the stomach to become distended, and its limits can be readily defined by percussion; this method often causes a considerable amount of discomfort to the patient, since carbonic acid gas has the property of causing spasm of the pylorus, and it has the further disadvantage that, in cases of hyperacidity of the stomach contents, the evolution of gas is prevented or becomes so small that it is not of any practical use. To obviate this it has been recommended to wash out the stomach with warm water before introducing the divided seidlitz powder; this, however, seems foolish, since if a stomach tube be passed it is better to directly pump air into the organ, and so avoid the pyloric spasm caused by the generation of carbonic acid gas.

The gastric diaphane consists of a flexible pipe, resembling a stomach tube, at the end of which is a small electric light carefully guarded, the electric wires being contained in the pipe. If this is passed into the patient's stomach in a dark room, and the electric light switched on, the area of the stomach will be clearly defined by the illumination which will be evident through the abdominal walls.

In the examination of the stomach contents it is usual to give what is known as a test breakfast; the best is that recommended by Ewald, and consists of a plain roll and half a pint of warm water or weak tea. This is given fasting in the early morning, and after an interval of an hour a stomach tube is passed and the remains of the breakfast expressed. This is filtered, and the filtrate is tested in the way to be described; of course, when vomiting is present the vomit can be filtered and the filtrate tested. The two principal tests are those for free hydrochloric acid and for lactic acid or lactates.

There are several tests for free hydrochloric acids, but the best is the following: A solution is made of 30 grains of phloroglaucin and 15 grains of vanillin in a fluid ounce of absolute alcohol; a few drops of this are placed on a china plate, and a drop of the stomach filtrate is placed close to this, so that the edges of the drops may coalesce; the plate is then warmed over a spirit lamp, and if any hydrochloric acid be present a red tinge will appear.

To test for lactic acid or lactates, a few drops of neutral ferric chloride solution should be added to about 3 drachms of 1 in 20 carbolic acid solution, and water added till an amethyst blue colour results; a few drops of the stomach filtrate is then added to this solution, which will produce a yellow colouration if lactic acid or lactates are present.

The capacity of the stomach can be measured by filling the stomach with water by means of the stomach tube, and then measuring the amount which can be obtained by expression; the normal capacity is about one and a half to two pints, but it may be increased to six or seven pints. Some estimate of the capacity of the stomach can also be formed from the amount of the vomit.

The rapidity of the stomach movements and the time taken in the passage of food out of the stomach can be tested by giving the patient five grains of salol and noting the time at which salicyluric acid appears in the urine. Salol is split up, in the presence of alkalis, into phenol and salicylic acid; but this change does not occur in acid media, and so cannot take place in the stomach, and so salicyluric acid cannot appear in the urine until salicylic acid has been absorbed from the intestinal walls. Under normal circumstances the acid appears in the urine within 40, or, at the most, 75 minutes after the salol has been swallowed. It can readily be recognised by the addition of neutral ferric chloride solution to the urine which produces a violet colour. To detect a mere trace a piece of filter paper should be dipped in the urine, and a drop of ferric chloride added; the edge of the drop becomes violet on warming in the presence of a mere trace of salicyluric acid. The presence of this acid in the urine does not last longer than 18 to 24 hours; but in cases of delay of the stomach movements it may be found as late as 30 or even 48 hours after being taken. If any be found after 30 hours we may take it that there is some abnormal delay.

The rapidity with which substances are absorbed from the stomach itself can be estimated by administering a dose of two grains of iodide of potassium in a cachet, and ascertaining the time at which iodine first appears in the saliva. The presence of iodine is demonstrated by moistening with saliva some filter paper which has been previously impregnated with starch and dried, and adding to this a drop of fuming nitric acid, when, if iodine be present, a blue colouration will appear. Under normal circumstances this will be found about fifteen minutes after the iodide has been swallowed, but where absorption is delayed it may not appear till after an interval of an hour or more.

I will give you an instance of the value of these chemical tests in cases of dilatation of the stomach, accompanied by a tumour in the pyloric region. The absence of free hydrochloric acid and the presence of lactic acid will be almost conclusive evidence in favour of carcinoma; if, on the other hand, there is abundance of free hydrochloric acid the tumour would probably prove to be an inflammatory thickening round a gastric ulcer.

The last method of examination is an exploratory laparotomy; at the present time the operation is practically devoid of risk, and should be performed in cases where a diagnosis cannot be accurately made by other means. I would not be understood to recommend laparotomy simply for the purpose of making a diagnosis, but rather in these cases where an uncertain diagnosis has been made, and then the operation is not merely exploratory, but in the event of the diagnosis being sustained a curative operation will be performed at the same time. Furthermore, it is within the knowledge of every surgeon that a large number of simple explorations are followed by complete relief of all the patient's symptoms, even when no tumour or abnormal condition is found, and finally in cases in which the tumour has appeared to be too far advanced for removal, the growth has completely disappeared after an exploratory operation.

